# Pemetrexed-Induced Pseudocellulitis: A Diagnostic Conundrum

**DOI:** 10.7759/cureus.52114

**Published:** 2024-01-11

**Authors:** Luke A Horton, Alexis B Lyons, Michael C Kwa, Marsha L Chaffins, Jesse Veenstra

**Affiliations:** 1 Dermatology, University of California Irvine, Irvine, USA; 2 Dermatology, Henry Ford Health System, Detroit, USA

**Keywords:** supportive onco-dermatology, oncodermatology, inpatient dermatology, complex medical dermatology, dermatology, cellulitus, drug reaction, infectious cellulitis, pseudocellulitis, pemetrexed

## Abstract

Pemetrexed, an anti-folate, antineoplastic agent, effectively treats various malignancies such as non-small cell lung cancer (NSCLC) and mesothelioma. Here, we report two cases of recurrent pemetrexed-induced lower extremity erythema and edema, one in a 60-year-old male and the other in a 47-year-old male, who were both treated for recurrent cellulitis on multiple occasions before finally being diagnosed with pemetrexed-induced pseudocellulitis (PIP), a rarely reported adverse effect. This is an important diagnostic pitfall for clinicians to be aware of, as early recognition may minimize patient morbidity and prevent unnecessary hospitalization and antibiotic use for presumed cellulitis.

## Introduction

Pemetrexed is a chemotherapy agent that prevents DNA synthesis by inhibiting three key enzymes: thymidylate synthase, glycinamide ribonucleotide formyltransferase, and dihydrofolate reductase. It treats multiple malignancies, including non-small cell lung cancer (NSCLC) and mesothelioma [1]. Common adverse effects include neutropenia, leukopenia, fatigue, dyspnea, renal toxicity, rash, conjunctivitis, nausea, and vomiting [1,2]. Here, we report two cases of pemetrexed-induced erythema and edema of the lower extremities that mimicked cellulitis, a phenomenon described as "pseudocellulitis" - a term characterizing a clinical presentation that is similar to cellulitis but is not a result of an infectious process [3-5]. Pseudocellulitis is a rare adverse effect associated with pemetrexed therapy that clinicians should be aware of, as early recognition will minimize patient morbidity, unnecessary hospitalizations, and antibiotic use.

## Case presentation

Case 1

**Figure 1:**
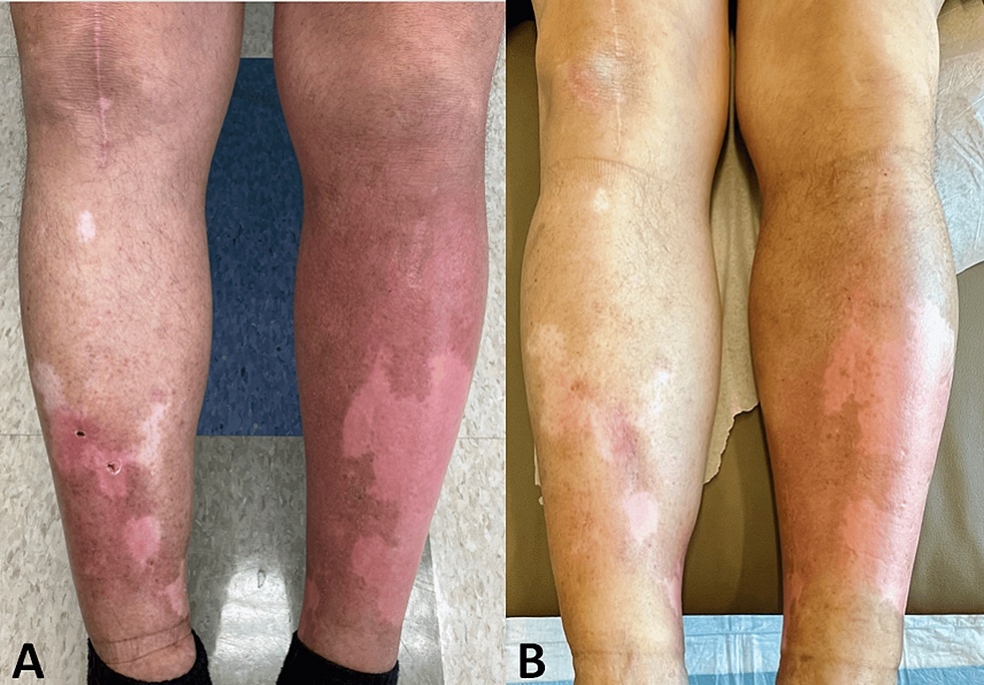
Warm, poorly defined erythematous patches on both shins without tenderness Asymmetric (left greater than right) erythema and swelling of the lower legs prior to (A) and following (B) the initiation of prednisone

A 60-year-old male with a four-year history of metastatic NSCLC receiving pemetrexed infusions (1.2 g every three weeks for the past eight months) presented to the ER with an episode of swelling and erythema of the lower extremities, suspected to be cellulitis, that began several days after his 12th pemetrexed infusion and had been ongoing for ten days. His primary care physician prompted him to go to the ER for intravenous (IV) antibiotics after his symptoms failed to improve with doxycycline and ciprofloxacin. On arrival, he was hemodynamically stable and denied having a recent fever or chills. Physical examination revealed warm, poorly demarcated erythematous patches on the bilateral shins without prominent tenderness (Figure 1A). There were also well-demarcated depigmented macules and patches on bilateral shins and buttocks due to unrelated chronic vitiligo that had appeared after the initiation of his cancer therapy. Routine labs were unremarkable, without an elevation in his white blood cell (WBC) count. This was the patient's third episode of bilateral lower extremity edema and erythema following a pemetrexed infusion in the past several months. However, this was the first time symptoms failed to improve after a short course of oral antibiotics. He ultimately received IV vancomycin and was discharged the next day.

Two days after his 13th pemetrexed infusion, the patient again developed an episode of erythema and edema of the lower extremities, resulting in admission to the hospital for recurrent cellulitis and treatment with vancomycin. Again, he experienced no fever, chills, or WBC count elevation. There were no actively draining wounds or systemic symptoms. Due to the recurrent nature of symptoms temporally associated with pemetrexed infusions, bilateral presentation, lack of exquisite tenderness, systemic symptoms, and laboratory findings to support cellulitis (ALT-70 score for cellulitis of 0), the patient was diagnosed with pemetrexed-induced pseudocellulitis (PIP) [6]. Furthermore, the Naranjo algorithm score, a questionnaire designed to assess the probability that a reaction is due to a drug or an alternative etiology, was calculated to be 7 - compatible with a probable adverse drug reaction to pemetrexed [7]. He started an eight-day 40 mg prednisone taper for the pseudocellulitis, with which the swelling and erythema improved (Figure 1B).

Given a diagnosis of PIP, dermatology recommended he forgo further antibiotics. Compression stockings and a course of prednisone 10 mg daily for a week following each pemetrexed infusion helped mitigate the swelling and erythema that occurred after each infusion (typically beginning two to three days later); however, it did not completely resolve his symptoms (Figure 1B). The patient understood that decreasing or discontinuing pemetrexed would likely lead to complete resolution, but he refused this option.

Case 2

A 47-year-old male with metastatic NSCLC undergoing therapy with loratinib and pemetrexed (1.1 g every three weeks over the past eight months) presented with progressive bilateral lower extremity edema and erythema that began several days after his 11th pemetrexed infusion. A month prior, the patient was hospitalized for a similar episode of lower extremity edema and pain and received broad-spectrum IV antibiotics without resolution of his symptoms. The patient was diagnosed with deep vein thrombosis in his left lower extremity and possible superficial thrombophlebitis in his right lower extremity by ultrasound and was discharged on therapeutic enoxaparin sodium.

**Figure 2:**
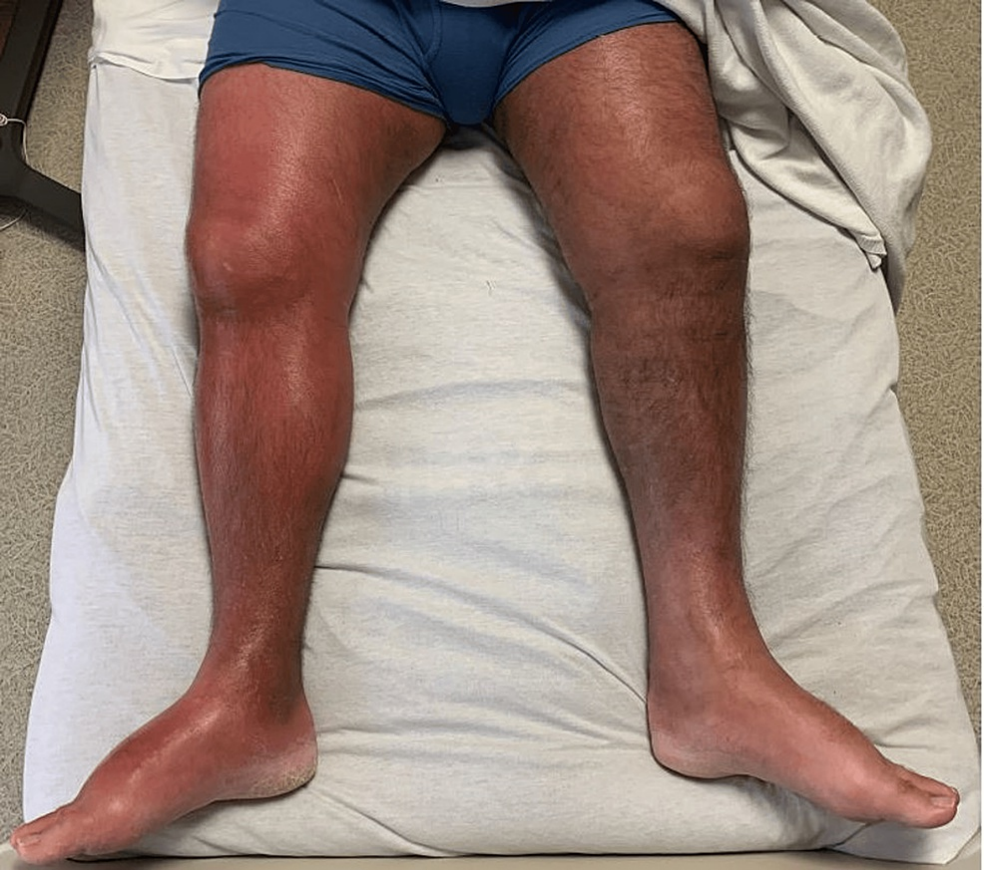
lower extremity edema Lower extremity edema, warmth, and a peau de orange appearance, along with ill-defined erythematous tender plaques extending from the foot to the right lower abdomen

**Figure 3:**
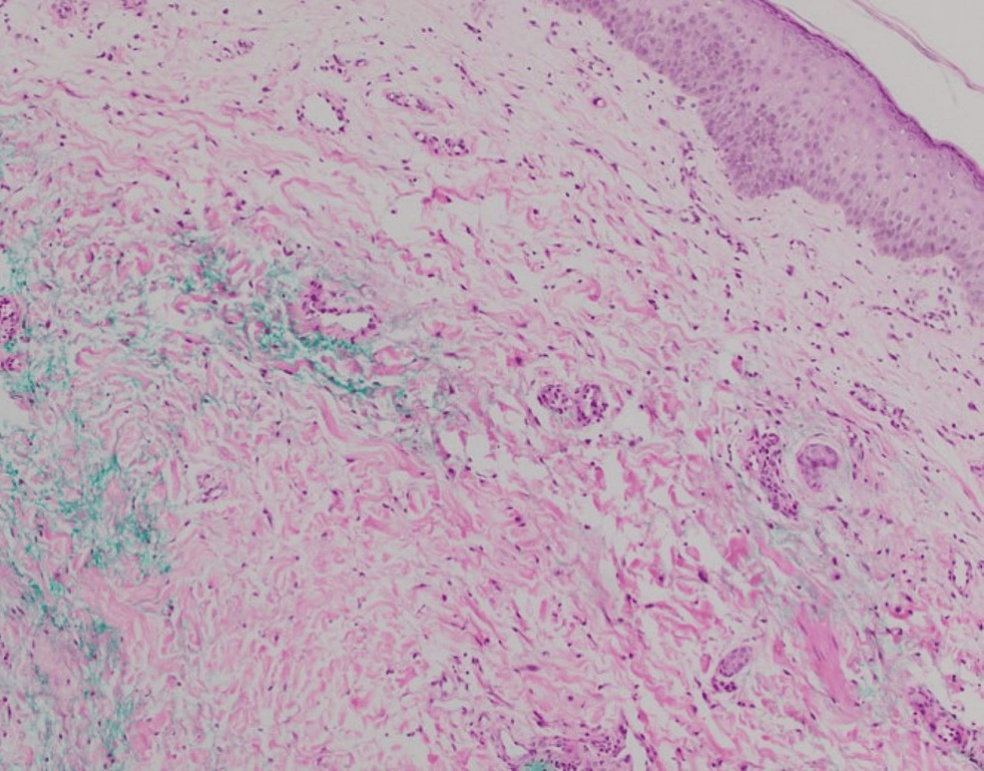
Histology of a punch biopsy Histology reveals dermal edema with a sparse interstitial infiltrate of neutrophils and occasional eosinophils

Despite appropriate anticoagulation, the erythema and edema began intensifying several days after his 12th pemetrexed infusion, affecting his right leg more than his left, with 10/10 pain, making walking difficult. The patient was afebrile on admission with a normal WBC count, and a repeat lower extremity duplex ultrasound found no deep venous thrombosis. He failed to show improvement with five days of broad-spectrum antibiotics (vancomycin and piperacillin/tazobactam) for suspected cellulitis. At this time, physical examination revealed marked bilateral (right greater than left) lower extremity edema, warmth, and a peau de orange appearance, with ill-defined erythematous tender plaques extending from the foot to the right lower abdomen (Figure 2). A punch biopsy revealed dermal edema with a sparse interstitial infiltrate of neutrophils and occasional eosinophils (Figure 3). A diagnosis of PIP resulted in the discontinuation of antibiotics, and the patient started a one-week course of prednisone 20 mg daily, 0.1% triamcinolone cream twice daily, and compression stockings with subsequent improvement. Unfortunately, the patient passed away from complications related to his malignancy one month after admission, despite the recommendation to co-administer oral steroids with pemetrexed infusions to help limit the symptomology of PIP.

## Discussion

Pseudocellulitis of the lower extremities is a rare adverse effect of pemetrexed therapy. It is often mistaken for infectious cellulitis despite a lack of fever or systemic signs of infection and, in some cases, associated tenderness [1,8]. Grade 1-2 peripheral edema (without erythema) occurs in approximately 4.5% of NSCLC patients receiving pemetrexed [9]. However, PIP should be considered when this edema presents with erythema [2,10-12]. The exact mechanism for PIP is unknown; however, it may be due to increased capillary protein leakage similar to that seen in docetaxel therapy [11]. Others suggest an etiology similar to eosinophilic cellulitis (Wells syndrome), where a hypersensitivity reaction occurs against pemetrexed accumulating in the skin [3].

Similar to the above cases, Lopes et al. reported a case of a 68-year-old man with stage IV NSCLC who developed asymmetric bilateral lower extremity erythema that mimicked cellulitis two weeks after his second pemetrexed infusion but was culture-negative and unresponsive to antibiotic therapy [13]. A biopsy revealed dermal edema, dilated dermal vessels, swollen endothelial cells, and a sparse perivascular infiltrate composed of lymphocytes, neutrophils, and eosinophils compatible with the changes in urticaria [13]. The dermatopathological features of PIP usually reveal non-specific inflammatory signs, often featuring edema, spongiosis, and lymphocytic and eosinophilic infiltrates, similar to our case [2-4,13]. Typically, the edema and redness of PIP resolve with discontinuation of pemetrexed therapy, but when discontinuation is not possible or preferred, systemic corticosteroids have been used prophylactically with favorable results [10,12].

When faced with localized erythema and edema of the lower extremities in a patient treated with pemetrexed, the differential diagnosis includes infectious cellulitis, erysipelas, eosinophilic cellulitis, and PIP. When differentiating PIP from cellulitis, providers must evaluate for recurrent nature, temporal association to pemetrexed infusions, and systemic signs of infection. Both of our patients began developing PIP after approximately six to eight months of infusions, with the reaction occurring two to three days after the infusions. However, other cases have reported shorter and longer intervals between pemetrexed exposure and symptom onset [13]. Involvement of the bilateral extremities or a lack of expected clinical response to appropriate antibiotic treatment for presumed cellulitis also suggests a non-infectious etiology [5,13,14]. Eosinophilic cellulitis can present abruptly as erythematous plaques and, like pseudocellulitis, may present without fever. However, pruritus is typically a dominant symptom in eosinophilic cellulitis, and the plaques may resolve with a greenish hue, which can be diagnostic [14]. Peripheral eosinophilia occurs in approximately 50% of patients, and tissue biopsy is consistent with eosinophilic cellulitis - with brisk interstitial infiltration of eosinophils in the mid to deep dermis with characteristic "flame figures" (eosinophilic granules surrounding collagen fibers) [14,15]. These histologic findings are not present in PIP [2-4,13].

Treatment of PIP includes halting or reducing the dose of pemetrexed (when possible), wearing compression stockings, and administering systemic corticosteroids. Currently, there is no standard steroid regimen for PIP. One case reported resolution of symptoms using 1 mg/kg of prednisone daily [13], and another used 20 mg of prednisone daily with symptomatic improvement [12]. Pretreatment with steroids before pemetrexed administration can be beneficial in cases where stopping pemetrexed therapy is unfavorable [5,8,13,15]. Antibiotics are not needed in the treatment of PIP.

## Conclusions

Here, we present two cases of PIP mistaken for infectious cellulitis. This is an important but rare adverse effect of pemetrexed that clinicians should be aware of, as diagnosis is often delayed, leading patients to suffer unnecessary pain, hospitalization, and antibiotic use. While no standard regimen for the treatment of PIP exists, it can be managed by coadministration of systemic steroids, decreased dosage, or discontinuation of the drug, though some may choose to treat the symptoms, depending on their severity.
